# Premature Valvular Heart Disease in Homozygous Familial Hypercholesterolemia

**DOI:** 10.1155/2017/3685265

**Published:** 2017-07-06

**Authors:** Akl C. Fahed, Kamel Shibbani, Rabih R. Andary, Mariam T. Arabi, Robert H. Habib, Denis D. Nguyen, Fady F. Haddad, Elie Moubarak, Georges Nemer, Sami T. Azar, Fadi F. Bitar

**Affiliations:** ^1^Division of Cardiology, Massachusetts General Hospital, Boston, MA, USA; ^2^Departments of Pediatrics and Adolescent Medicine, American University of Beirut Medical Center, Beirut, Lebanon; ^3^The Society of Thoracic Surgeons, Chicago, IL, USA; ^4^Department of Gastroenterology, Hepatology, and Nutrition, Boston Children's Hospital, Boston, MA, USA; ^5^Surgery, American University of Beirut Medical Center, Beirut, Lebanon; ^6^National LDL Apheresis Center, Dahr El Bacheck Government University Hospital, Dahr El-Bachek, Lebanon; ^7^Biochemistry and Molecular Genetics, American University of Beirut Medical Center, Beirut, Lebanon; ^8^Internal Medicine, American University of Beirut Medical Center, Beirut, Lebanon

## Abstract

Valvular heart disease frequently occurs as a consequence of premature atherosclerosis in individuals with familial hypercholesterolemia (FH). Studies have primarily focused on aortic valve calcification in heterozygous FH, but there is paucity of data on the incidence of valvular disease in homozygous FH. We performed echocardiographic studies in 33 relatively young patients (mean age: 26 years) with homozygous FH (mean LDL of 447 mg/dL, 73% on LDL apheresis) to look for subclinical valvulopathy. Twenty-one patients had evidence of valvulopathy of the aortic or mitral valves, while seven subjects showed notable mitral regurgitation. Older patients were more likely to have aortic valve calcification (>21 versus ≤21 years: 59% versus 12.5%; *p* = 0.01) despite lower LDL levels at the time of the study (385 versus 513 mg/dL; *p* = 0.016). Patients with valvulopathy were older and had comparable LDL levels and a lower carotid intima-media thickness. Our data suggests that, in homozygous FH patients, valvulopathy (1) is present across a wide age spectrum and LDL levels and (2) is less likely to be influenced by lipid-lowering treatment. Echocardiographic studies that focused on aortic root thickening and stenosis and regurgitation are thus likely an effective modality for serial follow-up of subclinical valvular heart disease.

## 1. Introduction

In 1952, Dr. Barr and colleagues from the New York Hospital-Cornell Medical Center reported a case of a 20-year-old man with xanthomata who died from severe nonrheumatic aortic stenosis [1]. Autopsy of the heart showed extensive atherosclerosis in the aorta and aortic valve. This case report was long before the discovery of echocardiography, CT scanning, or the LDL receptor. Subsequent studies from the 1960s through the mid-1980s reported patients with xanthoma dying from valvular heart disease. These led to defining familial hypercholesterolemia as a disease characterized by accelerated atherosclerosis which could result in severe supravalvular aortic stenosis and eventually death. On autopsy, lipid deposition, calcification, and inflammation of the aorta were documented [2–4]. Since then, scientific discovery has led to the cloning of the LDL receptor, development of lipid-lowering therapy, and improvement of diagnosis of valvular heart disease with echocardiography.

Familial hypercholesterolemia (FH) is a Mendelian disorder of abnormally high low-density lipoprotein (LDL) cholesterol. In the homozygous or severe form which occurs in a prevalence rate of around 1 in a million, LDL levels usually exceed 3 to 4 times the upper limit of normal. FH patients often present with external cholesterol depositions around their eyes (xanthelasmas) or joints (xanthomas) and are particularly prone to premature atherosclerotic cardiovascular disease (CVD) [5]. FH patients often require treatment with plasmapheresis in addition to lipid-lowering medications.

Clinical prognosis in FH focuses on prevalence of atherosclerotic CVD and its surrogate markers. However, a lesser studied impact of the disorder is the increased risk of developing valvular heart disease. Hypercholesterolemic valvulopathy remains a vague entity in FH patients [6]. Calcific aortic valve disease is the most described, with sparing literature detailing early mitral valve abnormalities [1, 2]. Multiple studies suggested that aortic valve calcification, best seen on computer tomography (CT) scan, is the earliest maker of aortic valve disease in FH patients [7, 8]. However, aortic valve calcification does not correlate with LDL levels or treatment in FH patients, and multiple randomized control clinical trials have shown no improvement of aortic valve calcification with lipid-lowering therapy [9–11]. There have been two cohort studies that looked at 10 and 39 homozygous FH patients, respectively [12, 13]. While patients in these studies had aortic valve calcification, there was high prevalence of aortic stenosis and regurgitation. Kawaguchi et al. studied 10 homozygous patients and 8 of them had aortic valve stenosis or regurgitation [12]. Kolansky et al. found that 8 out of 39 homozygous FH patients (21%) had mild-to-moderate aortic regurgitation [13]. The same study showed in longitudinal follow-up of a small group of the cohort that aortic regurgitation might be the earliest manifestation of valvulopathy in these patients and is associated with progression to clinically significant CVD [13]. These studies were limited by the small sample size and the heterogeneity of patients and testing done. The exact prevalence and distribution of subclinical valvular disease in this population remain unknown. No study has reported the complete echocardiographic findings on a sizeable cohort of relatively young, well phenotyped and genotyped, homozygous FH patients without previously known significant CVD.

We have previously reported genetic studies on the Lebanese FH Registry, which is one of the largest reported cohorts of homozygous FH in the literature [14, 15]. In this study, the same cohort of 33 patients is interrogated for evidence of subclinical premature valvular heart disease using echocardiography. We aimed to define the prevalence and characteristics of premature subclinical valvular heart disease in patients with severe FH using echocardiography and determine how factors such as LDL levels, plasmapheresis treatment, genotype, and age affect the disease.

## 2. Methods

### 2.1. Patients and Phenotypes

The study was approved by the Institutional Review Board of the American University of Beirut, and all patients or parents gave informed consent. Thirty-three Lebanese patients (mean age: 26; 51% female) with severe (clinically homozygous) hypercholesterolemia were recruited to the study from the Lebanese FH Registry as previously described. A detailed medical history, physical exam, and review of the medical records were performed by two clinicians. Fasting lipid panel measurements were obtained. This was drawn prior to an LDL apheresis session for those patients receiving this treatment. Genetic information on all subjects was also available from a prior study [14].

### 2.2. Cardiovascular Evaluations

Routine 2D echocardiography with color and Doppler was performed on all subjects in the study by two experienced cardiologists in echocardiography who were blinded to the clinical history of the patients. Two patients (67 and 76) had echocardiograms performed at a different institution and were reviewed by the same cardiologists. Valvulopathy was defined as aortic valve calcification, aortic root thickening, and any valvular stenosis or regurgitation that is mild or more. Aortic root thickening was defined as an anterior or posterior wall thickness of >2.2 mm as measured by M-mode at the end diastolic dimension of the aortic root walls at the sinuses and tubular portions from the longitudinal or short axis view. Aortic stenosis was defined as follows: mild (aortic jet velocity of 2.0–2.9 m/s and a peak systolic gradient and a mean gradient of <36 mmHg and <20 mmHg, resp.), moderate (aortic jet velocity of 3.0–4.0 m/s and a peak systolic gradient and a mean gradient of 36–64 mmHg and 20–40 mmHg, resp.), and severe (aortic jet velocity of >4 m/s and a peak systolic gradient and a mean gradient of >64 mmHg and >40 mmHg, resp.). Carotid duplex ultrasound and measurement of intima-media thickness were performed as previously described [16]. Ankle-brachial index (ABI) was measured for all patients on the right and left* dorsalis pedis* and* posterior tibialis*. Any ABI of ≤0.90 was considered abnormal based on the most recent ACC/AHA guidelines [17]. Presence of prior cardiovascular disease was defined as coronary artery disease requiring revascularization, nonembolic stroke, or carotid stenosis requiring surgery.

### 2.3. Statistical Analyses

Two group comparisons for summary measures were performed. Fisher's exact test was used to compare categorical variables. Continuous variables were not normally distributed and were compared using Wilcoxon's rank-sum test. A *p* value less than or equal to 0.05 was considered statistically significant. All analyses were performed using Stata/SE v.12.0 (Stata Corp., College Station, Texas).

## 3. Results

Thirty-three patients (52.5% female, mean age: 26 years) were studied. Patients in our cohort tended to have a rather severe phenotypic presentation as demonstrated by a mean LDL of 447 mg/dL and the fact that 73% were on plasmapheresis. Genetically, 82% had homozygous or compound heterozygous mutations in the* LDLR* or* ARH* genes, and the remainder had heterozygous mutations in these genes. Only 7 patients (21%, average age: 49 years) had known atherosclerotic CVD prior to enrollment in the study (Tables 1, 2, and 3). Subclinical vascular disease was also present with mean (average of right and left for all patients) carotid intima-media thickness (IMT) of 0.87 mm and 18% of patients having a reduced ankle-brachial index (ABI) (Table 1).

**Figure 1:**
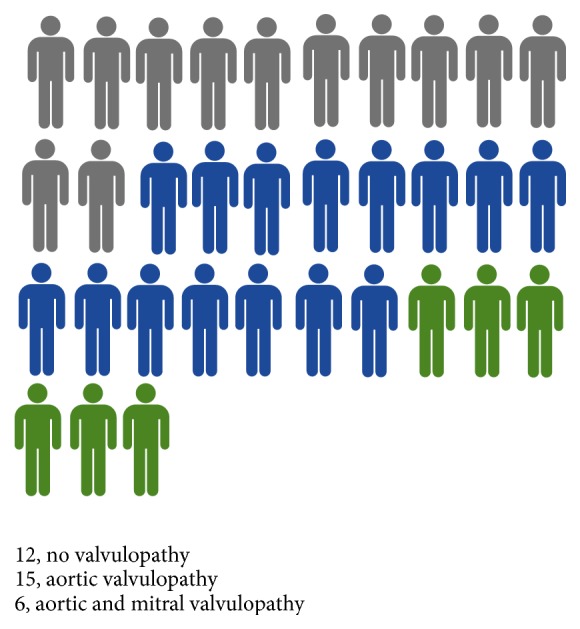
Prevalence of FH valvulopathy in homozygous FH. A diagram of the homozygous FH cohort showing patients without valvulopathy (grey), with aortic valvulopathy (blue), and with both aortic and mitral valvulopathies (green).

Despite the young age of the cohort, 64% (21 subjects) had subclinical valvular heart disease on echocardiography (Table 1 and Figure 1). Five out of 7 patients with known atherosclerotic CVD had also valvular findings on echocardiography and two did not. Excluding the 7 patients with known atherosclerotic CVD, 61.5% (16 out of 26) had subclinical valvular heart disease on echocardiography. All valvular pathology involved the left-sided heart valves. Of the 21 subjects with valvulopathy, all had one or more aortic pathologies, while 6 had concomitant mitral regurgitation (Figure 1). Among the aortic valve pathologies, root thickening was the most common (14 out of 21), followed by aortic valve stenosis (13 out of 21) and aortic valve calcification (12 out of 21) (Table 1). All patients with aortic stenosis (AS) had valvular aortic stenosis. These included three patients with moderate AS and ten patients with mild AS. Nine patients (43% of valvulopathy patients) had aortic root thickening or valve regurgitation or stenosis in the absence of aortic valve calcification, and three patients (14, 6, and 17) had calcification without other valvular or root pathologies (Table 3). These patients were distributed also across a wide age spectrum.

The cohort had a median age of 24 years. The younger half of the cohort (≤21 years) had significantly higher mean LDL levels compared to the older half (>21 years). All seven patients with known cardiovascular disease were also >21 years old. The older patients were also more likely to have valvular heart disease (82%) as compared to 33% in the younger patients (*p* = 0.032). The difference was also statistically significant for aortic valve calcification (58% versus 12%, *p* = 0.01) (Table 1).

Patients with valvulopathy (*n* = 21) were on average older than those without valvulopathy (*n* = 12) (31 versus 17 years, *p* = 0.02) and were more likely to be on apheresis (85% on apheresis for an average duration of 7 years versus 50% for an average of 4 years) (Table 2). Table 3 shows the patients ranked by increasing age. Older patients tend to have more diffuse disease involving the aortic valve, aortic root, mitral valves, and carotid. Finally, patients with valvular disease had a mean IMT (0.62 mm) that was smaller than that of those without (1.01 mm, *p* = 0.035) (Table 2).

## 4. Discussion

In this cohort study of 33 relatively young patients with severe FH, the major findings were as follows: (1) subclinical FH valvulopathy was present in 64% of patients on echocardiography; (2) aortic root thickening, aortic stenosis, aortic regurgitation, and mitral regurgitation occurred commonly in severe FH patients and sometimes in the absence of aortic valve calcification; (3) older patients were more likely to have aortic valve calcification despite lower LDL-C levels; and (4) patients with valvulopathy were older and were more likely to be on LDL apheresis but with lower intima-media thickness compared to patients who did not have valvulopathy.

The findings from this study expand our understanding of the subclinical premature occurrence of valvular heart disease in severe FH. Besides several case reports, there have only been two published echocardiography studies of 39 and 10 severe FH patients, respectively [12, 13]. Both studies highlighted FH valvulopathy as a distinct feature from aortic valve calcification seen in heterozygous FH patients, often using computed tomography [7]. FH valvulopathy is characterized by involvement of the left-sided valves, primarily the aortic valve. Kolansky et al. concluded that aortic regurgitation is the most prevalent and earliest sign of FH valvulopathy, and it was associated with presence of angiographic coronary stenosis [13]. In a smaller cohort, Kawaguchi et al. reported that 8 out of the 10 homozygous FH patients had aortic valve stenosis [12]. No significant involvement of mitral valve disease in homozygous FH patients was reported in the two major cohorts cited or prior case reports. However, in a group of 39 heterozygous FH patients, the prevalence of mitral regurgitation was 41% but was not significantly different from a control group [12]. In our study, we confirm previous findings that, in severe FH, valvulopathy on echocardiography involved regurgitant and stenotic aortic valvular phenotypes without necessarily calcification. In contrast to the prior two cohorts, we show that valvulopathy phenotype is heterogeneous and is not limited to aortic regurgitation or stenosis. In fact, the single most common finding was aortic root thickening in 42% of patients (*n* = 14), while mitral regurgitation was seen in 21% of patients (*n* = 6). Overall, 64% of patients (*n* = 18) had at least one abnormal valve finding on echocardiography. All six patients who had mitral regurgitation also had aortic valvulopathy.

Most of the studies investigating valvular heart disease in FH have focused on aortic valve calcification. In one study, cardiac CT was used to screen a large cohort of older heterozygous FH patients for aortic valve calcification and found that it occurred in 41% of cases compared to 21% of controls (*p* < 0.001) [7]. In that study, aortic valve calcification was independently associated with age, LDL levels, and diastolic blood pressure and was predicted strongly by the presence of a loss of function mutation in the LDL receptor gene (OR: 4.81, *p* < 0.001) [7]. Other studies reported prevalence of aortic valve calcification approaching 100% in homozygous FH [18, 19]. In this study, 43% of patients with valvulopathy did not have aortic valve calcification and three had aortic valve calcification without evidence of other pathologies on echocardiography. Echocardiogram is less sensitive than cardiac CT scan in detecting aortic valve calcification and as such it is possible that some of the patients in our study have calcifications that are not detected on echocardiography.

Aortic stenosis in severe FH was one of the first cardiovascular phenotypes to be described in several case reports prior to echocardiography based on clinical exam, hemodynamic assessment, and autopsy [1–4]. These severe presentations, in an era prior to LDL apheresis or statins, were a good depiction of the natural history of disease. Severe supravalvular aortic stenosis continues to be seen on echocardiography on presentation of untreated severe FH children [20]. In an older study form 1989 describing 18 patients with homozygous FH, supravalvular stenosis was also common, detected in 23% (3/13) of patients by echocardiography and 54% (6/11) of the cohort by catheterization [21]. With LDL-lowering with both lipid-lowering therapy and LDL apheresis, the severity of AS is less but is still higher than what you would expect in otherwise healthy young population [12, 13]. In our cohort, 13 patients (40%) had aortic stenoses which were all mild with the exception of patient 1 (42 years old) who had moderate AS. He also has severe carotid stenosis and coronary artery disease. Although all aortic stenoses were valvular, it is likely that aortic root thickening reflects a milder form of supravalvular cholesterol deposition. The patients with aortic stenosis were distributed across wide age spectrum, LDL levels, and duration of lipid-lowering therapy. The low sample size does not allow making conclusions regarding specific characteristics of those patients with AS. Similarly, 8 patients (24%) had aortic regurgitation, five of which also had concomitant stenosis. This prevalence is lower than the one reported by Kolansky et al. [13]. Relying on aortic regurgitation as the only earliest indicator of valvular pathology in this population would likely miss a large portion of FH patients with subclinical valvular heart disease.

Aortic root thickening was the most prevalent finding in our patients (42%) and often coincided with not only aortic regurgitation but also calcified valve and aortic stenosis. Root thickening per se on echocardiography had not been described in the two prior cohorts. Kawaguchi described cusp thickening as a result of lipid accumulation as well as infiltration of inflammatory cells, which eventually leads to regurgitation [6]. We believe that a similar process occurs in the aortic root and is likely the earliest manifestation of supravalvular aortic stenosis which we see in our study but it also has been well described in the multiple untreated case reports in the early literature [3, 4, 20, 22]. Also atheromatous plaques in the root and ascending aorta have been described in heterozygous FH patients with lower LDL levels [12]. Additionally, the internal diameter of the supravalvular aortic ridge has been shown to be smaller in FH patients compared to controls, with reduced distensibility [12]. The hemodynamics of the aortic root have been further studied, showing that FH patients have increased aortic stiffness and reduced distensibility [23].

Mitral valvulopathy is less described in FH patients. Eight patients (24%) had mitral regurgitation in our cohort of severe FH patients. These included at least three young patients (patient IDs 3, 11, and 68 with ages of 19, 21, and 31 years, resp.) without a history of moderate or above AS or known coronary artery disease to justify ischemia or cardiomyopathy as a cause of the mitral regurgitation. While mitral regurgitation in this population could occur secondary to ischemia and cardiomyopathy, our data do suggest a primary valvulopathy of the mitral valve. We hypothesize the three potential mechanisms, silent subclinical ischemia of the papillary muscles, calcification of the mitral annulus, or hemodynamic strain on the left ventricle, as a consequence of the decreased distensibility of the aorta.

Limited studies suggested the presence of molecular mechanisms of FH valvulopathy independent of LDL Levels but related to other downstream effects of the genetic mutation [8]. We are unable, however, in our case to see any difference between patients with homozygous (complete loss of LDL receptor function) and nonhomozygous mutations. This could be limited by the small power of our study to detect such differences which will always be challenging with severe FH given the low prevalence of disease. This is consistent with our prior findings that LDL levels and not mutation status predict carotid intima-media thickness in FH [16]. However, patients with valvulopathy, despite being older and having comparable LDL levels than those without valvulopathy, had a significantly lower intima-media thickness. This suggests that valvulopathy, unlike intima-media thickness, is less likely to correlate with fluctuations in LDL levels, that is, reduction of intima-media thickness without resolution of valvular findings after getting apheresis. This remains, however, to be proven by longitudinal studies. It is possible that patients with FH are exposed to high LDL very early on in life, and this exposure is what triggers downstream molecular pathways leading to irreversible structural valvular changes. A factor of LDL levels multiplied by age or duration of exposure (area under the curve) seems to be the major determinant of valvulopathy.

This study has multiple limitations. First it is a cross-sectional study and as such using age and duration of treatment can only suggest trends in progression of disease; conclusions regarding progression of disease could only be made with longitudinal follow-up. Second, the study did not look specifically at calcifications using cardiac CT to determine how this correlated with echocardiographic findings. Finally, lipoprotein-a (LPA) levels were not measured as part of the study. Recent studies have shown that genetically determined elevation in LPA levels is associated with aortic stenosis in the general population [24] and is also an independent risk factor of aortic valve calcification in FH [25].

Findings of very high prevalence of subclinical valvular heart disease in a young population of severe FH patients have important clinical implications on the management of these patients. While this study is cross-sectional, we know from the natural history of disease that severe symptomatic supravalvular aortic stenosis occurs in untreated patients. Also limited longitudinal data has shown that at least subclinical aortic regurgitation correlates with clinically significant cardiovascular disease [13]. Based on this, it is reasonable to recommend echocardiography at diagnosis and serially every 1-2 years on all patients with this severe FH.

## 5. Conclusion

We show that subclinical valvular heart disease could be detected in a very high proportion of severe FH patients using echocardiography. We also characterize FH valvulopathy as a combination of one or more of aortic root thickening, aortic valve regurgitation, stenosis or calcification, and mitral regurgitation which progresses with age on treatment but at a remarkably accelerated rate.

## Figures and Tables

**Table 1:** Demographic and clinical characteristics.

Characteristics	All patients (*N* = 33)	≤21 (*N* = 16)	>21 (*N* = 17)	*p* value
Age, mean (SD)	26.12 (16.40)	13.56 (4.97)	37.94 (14.45)	
Female, % (*N*)	51.5 (17)	68.75 (11)	35.29 (6)	0.084
LDL (mg/dL), mean (SD)	447.45 (151.2)	513.40 (114)	385.30 (158.31)	0.016
HDL (mg/dL), mean (SD)	45.43 (26.88)	46.94 (33.26)	44.02 (20.10)	0.87
Genetically homozygous, % (*N*)	57.58 (19)	68.75 (11)	47.06 (8)	0.30
Apheresis, % (*N*)	72.73 (24)	62.50 (10)	82.35 (14)	0.26
Xanthoma, % (*N*)	93.94 (31)	93.75 (15)	94.12 (16)	1
Xanthelasma, % (*N*)	39.39 (13)	25 (4)	52.94 (9)	0.16
Arcus senilis, % (*N*)	42.42 (14)	37.50 (6)	47.60 (8)	0.73
IMT, mean (SD)	0.87 (0.57)	0.67 (0.45)	1.06 (0.62)	0.084
Reduced ABI^*α*^, % (*N*)	18.18 (6)	6.25 (1)	29.41 (5)	0.17
Clinical CVD, % (*N*)	21.21 (7)	0 (0)	41.18 (7)	0.0072
Valvulopathy^*β*^, % (*N*)	63.6 (21)	33.33 (7)	82.35 (14)	0.032
Aortic valve disease, % (*N*)	63.63 (21)	43.75 (7)	82.35 (14)	0.032
Calcification, % (*N*)	36.36 (12)	12.50 (2)	58.82 (10)	0.01
Root thickening, % (*N*)	42.42 (14)	25 (4)	58.82 (10)	0.08
Regurgitation, % (*N*)	24.24 (8)	12.50 (2)	35.29 (6)	0.22
Stenosis, % (*N*)	39.39 (13)	37.50 (6)	41.18 (7)	1
Mitral valve regurgitation, % (*N*)	21.21 (7)	12.50 (2)	23.53 (4)	0.66

LDL: low-density lipoprotein cholesterol; HDL: high-density lipoprotein; ABI: ankle-brachial index; IMT: intima-media thickness; CVD: cardiovascular disease. ^*α*^Reduced ABI was defined as any ABI (among right or left dorsalis pedis or posterior tibialis) that is ≤0.9. ^*β*^Valvulopathy includes patients with calcifications/aortic root thickening only (*N* = 4, 12.1% of the cohort) or valvular stenosis or regurgitation with or without calcifications (*N* = 17, 51.5% of the cohort).

**Table 2:** FH patients with and without valvulopathy.

Characteristics	Valvulopathy (*N* = 21)	No valvulopathy (*N* = 12)	*p* value
Age, mean (±SD)	31 (14.41)	17.5 (16.66)	0.020
Female, % (*N*)	52.38 (11)	50 (6)	1
LDL (mg/dL), mean (±SD)	436.7 (154.85)	466.25 (149.21)	0.50
HDL (mg/dL), mean (±SD)	39.46 (18.14)	55.89 (36.29)	0.13
Genetically homozygous, % (*N*)	85 (18)	75 (9)	0.64
Apheresis, % (*N*)	85.7 (18)	50 (6)	0.044
Apheresis years, mean (±SD)	7.33 (4.46)	3.92 (5.32)	0.072
IMT, mean (±SD)	0.62 (0.31)	1.01 (0.64)	0.035
Reduced ABI, % (*N*)	28.57 (6)	16.67 (2)	0.68
Clinical CVD, % (*N*)	38.10 (8)	33.33 (4)	1

**Table 3:** Vascular and Valvular Heart Disease in FH Patients Ranked by Age.

Patient ID	Age (years)	LDL (mg/dL)	Genotype	Homozygous	Apheresis (years)	Previously known CVD	IMT	Vascular disease	Valvular heart disease
49	6	603	*LDLR* C681X	Yes	0		0.4	Plaque	
80	7	579	*LDLR* C681X	Yes	0		0.45		
64	8	439	*LDLRAP* Q136X	Yes	0		0.5		
22	9	644	*LDLR* C681X	Yes	2		0.6		
50	10	565	*LDLR* C681X	No	0		1.45	CS, plaque	
61	10	544	*LDLR* C681X	Yes	0		0.45	CS, plaque	
77	10	224	*LDLR* C681X	No	0		0.45		
7	15	451	*LDLR* C681X	*Yes*	9		0.55		mild AS (MG 16, PG 33), ART
16	15	387	*LDLRAP* Q136X	Yes	2		0.55		
20	15	500	*LDLR* C681X	Yes	9		0.6		
2	16	455	*LDLR* C681X	Yes	8		0.55		mild AR, mild AS (MG 10, PG 27)
12	17	558	*LDLR* C681X	Yes	9		0.5		mild AS (MG 11, PG 26), ART
3	19	652	*LDLR* C681X	Yes	11		0.55	Plaque	mild AR, mild AS (MG 9, PG 24), mild MR
14	19	420	*LDLRAP* Q136X	Yes	2		0.5		AoVC
6	20	544	*LDLR* C681X	Yes	12		2.1	Plaque	mild AS (MG 16, PG 34), ART
11	21	649	*LDLR* I451T	Yes	10		0.5		mild AS (MG 19, PG 35), mild MR, ART, AoVC
15	24	527	*LDLRAP* Q136X	Yes	2		0.95	Plaque	mild AS (MG 11, PG 24), ART
8	25	582	*LDLRAP* Q136X	No	9		0.45		
26	25	638	*LDLR* C681X	Yes	7		1.3	CS, plaque	mild AR
23	27	541	*LDLR* C681X	Yes	13		1.8	Plaque	mild AR, moderate AS (MG 34, PG 64), AoVC, ART
67	28	390	*LDLRAP* Q136X	Yes	0	MI	0.45		mild AS, ART
21	29	495	*LDLR* C681X	Yes	5		1.05		mild AR, ART
75	30	219	*LDLR* C681X	No	10	Stroke	0.5		
25	31	545	*LDLR* C681X	Yes	8		2.05	CS, plaque	mild AR, moderate AS (MG 40, PG 64), mild MR, AoVC, CMV, ART
66	31	264	*LDLRAP* Q136X	Yes	0		0.5		AoVC
68	31	245	*LDLRAP* Q136X	Yes	0		1.5	CS, plaque	mild MR, AoVC
17	32	505	*LDLRAP* Q136X	Yes	10		0.55		AOVC
1	42	413	*LDLR* C681X	Yes	9	MI, CEA	2.1	CS, plaque	mild AR, moderate AS (MG 40, PG 64), moderate MR, AoVC, ART
30	46	348	*LDLR* C681X	Yes	6		0.8	CS, plaque	AoVC, ART
29	57	202	*LDLR* C681X	Yes	16	MI	0.5	Plaque	mild AS (MG 20, PG 36), AoVC, ART
76	57	158	*LDLR* C681X	No	7	MI, CEA, stroke	0.5	CS	mild AS, AoVC, CMV, ART
13	65	169	*LDLR* C681X	No	10	MI, CEA	2	CS, plaque	mild AR, moderate MR, AoVC, CMV, ART

CEA: carotid endarterectomy; MI: myocardial infarction; CS: carotid stenosis; AS: aortic stenosis; ART: aortic root thickening; AR: aortic regurgitation; MR: mitral regurgitation; AoVC: aortic valve calcification; CMV: calcified mitral valve; MG: mean gradient (in mmHg); PG: peak gradient (in mmHg).
